# Supplementary material for: AstroECP: towards more practical electron channeling contrast imaging
Source: J Appl Crystallogr. 2026 Feb 27;59(Pt 2):530–51. doi: 10.1107/S1600576726000567 (PMC13060471; doi:10.1107/S1600576726000567)
Supplement: Supplementary file 1 [file j-59-00530-sup1.pdf]

JOURNAL OF APPLIED CRYSTALLOGRAPHY

Volume 59 (2026)

Supporting information for article:

AstroECP: towards more practical electron channeling contrast imaging

M. Haroon Qaiser, Lukas Berners, Robin J. Scales, Tianbi Zhang, Martin Heller, Jiří Dluhoš, Sandra Korte-Kerzel and T. Ben Britton

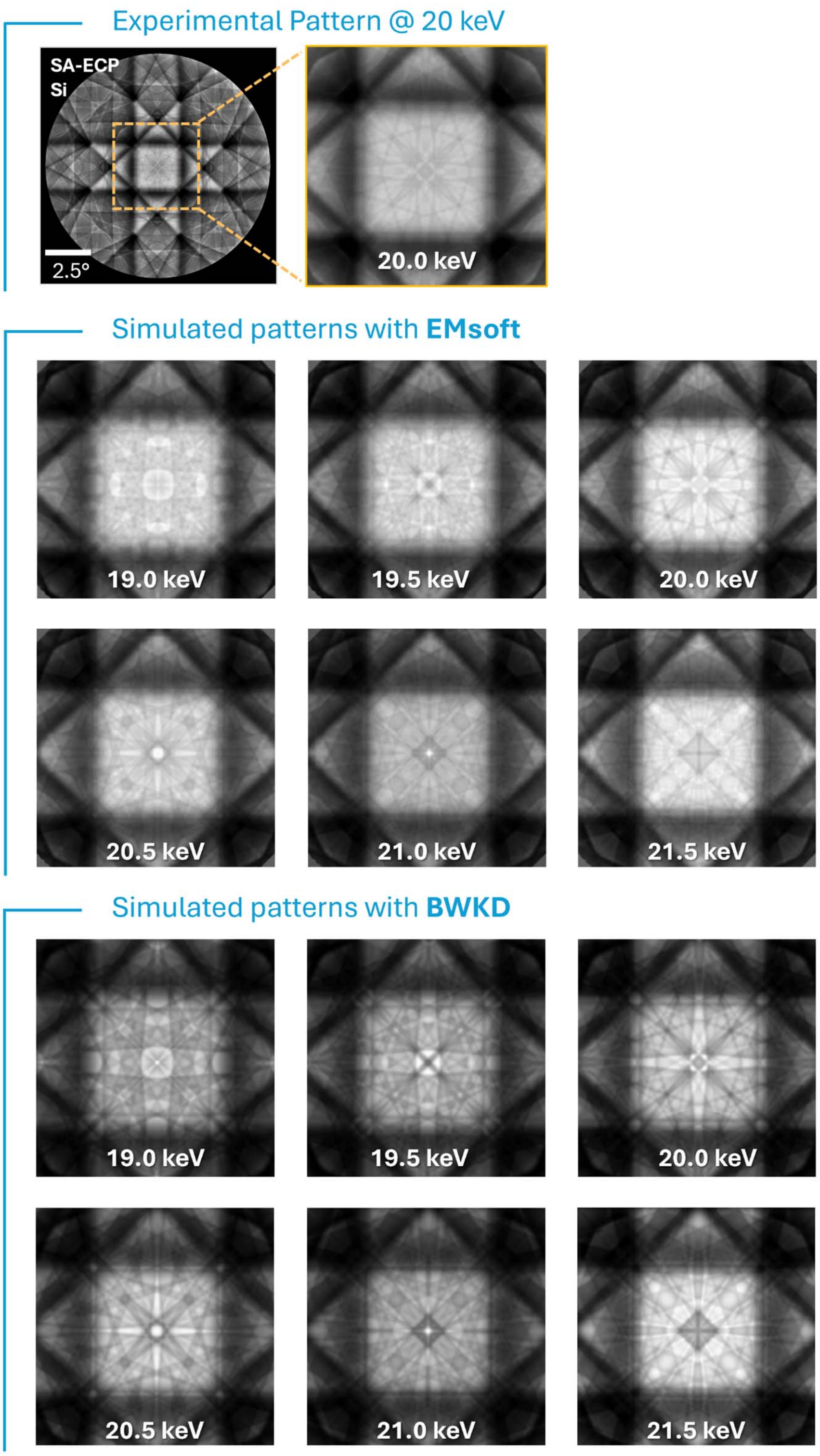

Figure S1 Experimental vs. simulated SA-ECP at various beam energies. Note the strong dependence of voltage on the geometry of high spatial frequency features inside the zone axis

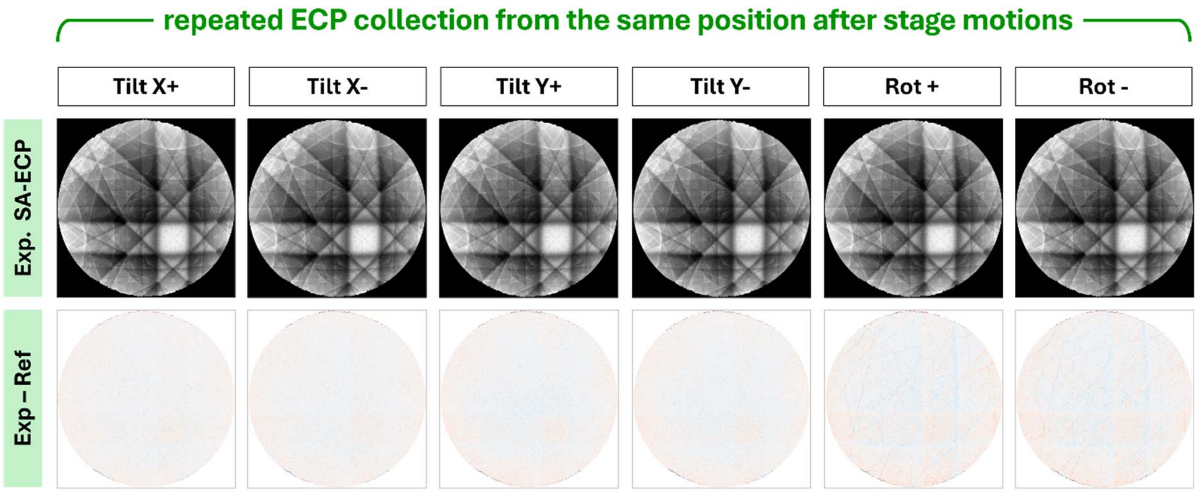

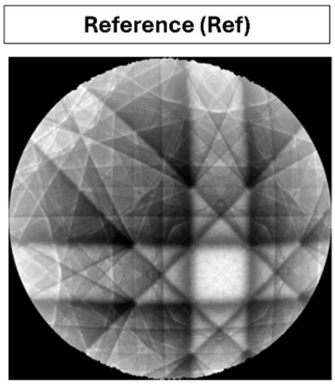

	Euler angles			DD	Δg from Ref, deg
	ϕ_1	Φ	ϕ_2		
Ref	42.00	3.38	3.21	3.85	0.000
Tilt X+	42.00	3.38	3.20	3.85	0.004
Tilt X-	42.00	3.38	3.21	3.85	0.001
Tilt Y+	42.00	3.38	3.19	3.85	0.019
Tilt Y-	42.00	3.38	3.20	3.85	0.001
Rot +	42.05	3.36	3.21	3.86	0.059
Rot -	42.02	3.35	3.23	3.85	0.055

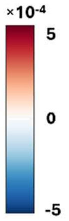

Figure S2 Evaluation of stage backlash through repeated SA-ECP collection from the same position after different stage motions. Top row shows experimental SA-ECPs after stage motion, second row shows their intensity difference plots with respect to reference SA-ECP. The orientation data is also tabulated, with maximum misorientation Δg of 0.059° was recorded in rotation.
